# HIV-1 Fusion with CD4+ T cells Is Promoted by Proteins Involved in Endocytosis and Intracellular Membrane Trafficking

**DOI:** 10.3390/v11020100

**Published:** 2019-01-25

**Authors:** Mariana Marin, Yulia Kushnareva, Caleb S. Mason, Sumit K. Chanda, Gregory B. Melikyan

**Affiliations:** 1 Department of Pediatric, Division of Infectious Diseases, Emory University School of Medicine, 2015 Uppergate Drive, Atlanta, GA 30322, USA; mmarin@emory.edu (M.M.); cs.mason@outlook.com (C.S.M.); 2 Functional Genomics Center, La Jolla Institute for Allergy and Immunology, 9420 Athena Circle, La Jolla, CA 92037, USA; yulia@lji.org; 3 Sanford Burnham Prebys Medical Discovery Institute, 10901 North Torrey Pines Road, La Jolla, CA 92037, USA; schanda@sbpdiscovery.org; 4 Children’s Healthcare of Atlanta, Atlanta, GA 30322, USA

**Keywords:** HIV, virus fusion, endocytosis, shRNA screen, cell fusion, membrane trafficking

## Abstract

The HIV-1 entry pathway into permissive cells has been a subject of debate. Accumulating evidence, including our previous single virus tracking results, suggests that HIV-1 can enter different cell types via endocytosis and CD4/coreceptor-dependent fusion with endosomes. However, recent studies that employed indirect techniques to infer the sites of HIV-1 entry into CD4+ T cells have concluded that endocytosis does not contribute to infection. To assess whether HIV-1 enters these cells via endocytosis, we probed the role of intracellular trafficking in HIV-1 entry/fusion by a targeted shRNA screen in a CD4+ T cell line. We performed a screen utilizing a direct virus-cell fusion assay as readout and identified several host proteins involved in endosomal trafficking/maturation, including Rab5A and sorting nexins, as factors regulating HIV-1 fusion and infection. Knockdown of these proteins inhibited HIV-1 fusion irrespective of coreceptor tropism, without altering the CD4 or coreceptor expression, or compromising the virus’ ability to mediate fusion of two adjacent cells initiated by virus-plasma membrane fusion. Ectopic expression of Rab5A in non-permissive cells harboring Rab5A shRNAs partially restored the HIV-cell fusion. Together, these results implicate endocytic machinery in productive HIV-1 entry into CD4+ T cells.

## 1. Introduction

As an enveloped virus, HIV-1 delivers its genome to the cytoplasm by fusing the viral membrane to the host cell membrane. This critical step of infection is driven by the HIV-1 envelope glycoprotein (Env) that sequentially binds to CD4 and coreceptors, CXCR4 or CCR5. The formation of the ternary Env-CD4-coreceptor complex induces refolding of the transmembrane gp41 subunit into the 6-helix bundle structure that promotes the merger of viral and cellular membranes (reviewed in [1,2,3,4]). Whether HIV-1 enters cells by fusing with the plasma membrane or with endosomes is a subject of active debate. Due mainly to its pH-independent entry, early studies suggested that productive HIV-1 fusion occurs at the plasma membrane (reviewed in [5]). Additional arguments against endocytic entry of HIV-1 into dendritic cells and CD4+ T cells are based on a combination of pharmacologic and genetic interventions, along with the use of reduced temperature that is permissive for endocytosis, but not for viral fusion [6,7,8,9,10]. However, accumulating evidence from several groups, including ours, supports the notion that productive HIV-1 entry into different cell types proceeds through endocytosis and virus-endosome fusion [9,11,12,13,14,15,16,17,18,19,20].

The reliance on indirect assays to deduce the sites of HIV-1 entry into cells in most published studies has confounded the interpretation of results. To pinpoint the cellular sites of HIV-1 entry, we have previously visualized single virus fusion in live HeLa-derives cells and found that fusion occurred with endosomal compartments [11,14]. Unfortunately, CD4+ T cells are not readily amenable to imaging of single virus hemifusion and fusion, which would enable direct assessment of the sites of virus entry. We have therefore explored alternative means to determine the role of endocytosis in HIV-1 fusion with these and other cell types by designing a dual-readout assay to measure free virus-cell fusion and virus-mediated cell-cell fusion in the same sample [13]. Free HIV-1 entry and virus-mediated cell-cell fusion, occurring as a result of virus fusion at the plasma membrane, exhibited drastically different efficiencies and requirements for actin dynamics in adherent cells and in CEM cells [13]. These results highlight the differences between the major HIV-1 entry pathway into these cells and rare fusion events for virions confined to the plasma membrane.

Here, we probed whether endocytic trafficking contributes to HIV-1 fusion with CD4+ T cells by performing a targeted short hairpin (shRNA) screen for host factors involved in intracellular membrane transport. Although previous genetic screens have identified several factors involved in endocytosis and vesicle transport [21,22,23,24,25] as essential for HIV-1 infection, the reliance on long-term HIV-1 replication assays makes it difficult to assess a role in the HIV-1 fusion step. We therefore used a direct HIV-cell fusion assay as a readout for the targeted genetic screen in CEM cells and identified several regulators of endocytic trafficking that were involved in HIV-1 fusion. Our results demonstrate that inhibition of HIV-1 fusion with T cells depleted of endocytic trafficking factors is not caused by the altered expression of CD4 or coreceptors, or by the compromised ability to fuse with the plasma membrane. The finding that unperturbed intracellular transport processes play a significant role in productive HIV-1 entry into a CD4+ T cell line supports the notion that a major fraction of viruses infects these cells by fusing with endosomes.

## 2. Materials and Methods

### 2.1. Cell Lines, Reagents and Plasmids

CEM-NKR-CCR5-Luc cells (donated by Drs. J. Moore and C. Spenlehauer [26]) and TZM-bl cells expressing CD4, CXCR4 and CCR5 (donated by Drs. J.C. Kappes and X. Wu [27]) were from the NIH AIDS Research and Reference Reagent Program. HEK293T/17 (human embryonic kidney) cells were purchased from the ATCC (Manassas, VA, USA). HEK293T/17 and TZM-bl were cultured in DMEM (Mediatech, Manassas, VA, USA) containing 10% heat inactivated fetal bovine serum (FBS, Sigma-Aldrich, St. Louis, MO, USA), and 100 units/mL of penicillin-streptomycin (Gemini Bio-Products, Sacramento, CA, USA). The growth medium for HEK293T/17 and CEM-NKR-CCR5-Luc cells was supplemented with 0.5 and 0.8 mg/mL of G418 sulfate (Mediatech), respectively.

The C52L recombinant peptide [28] was a gift from Dr. Min Lu (New Jersey School of Medicine). AMD3100, mouse anti-CD4 and mouse anti-tubulin were purchased from Sigma-Aldrich. Rabbit anti-Rab5A, mouse anti-clathrin heavy chain (CLTCL1), goat anti-SNX3 and anti-SNX10 antibodies were from Santa Cruz Biotechnology (Dallas, TX, USA). Rabbit anti-Rab7A was from Cell Signaling Technology (Danvers, MA, USA). The CCF4 acetoxymethyl ester (CCF4-AM) β-lactamase substrate (GeneBLAzer in vivo detection kit) was from Invitrogen (Carlsbad, CA, USA). Celltraker^TM^ green (CMFDA), Celltraker^TM^ orange (CMRA), Live-imaging buffer and OPTI-MEM were from Life Technologies (Grand Island, NY, USA).

The HXB2 envelope glycoproteins (Env) in pCAGGS plasmid was a gift from Dr. J. Binley (Torrey Pines Institute, CA) [29]. The packaging HIV-1 pR8ΔEnv vector was from Dr. D. Trono (University of Geneva, Switzerland). The following expression vectors were received from the NIH AIDS Research and Reference Reagent Program: HIV-1 BaL.26 Env (donated by Dr. J. Mascola [30]), pMM310 expressing BlaM-Vpr (donated by Dr. M. Miller [31], psPAX2 lentiviral packaging vector (donated by Dr. D. Trono), and pcRev vector (donated by Dr. B.R. Cullen [32]). pMDG-VSVG plasmid expressing VSV-G was a gift from Dr. J. Young (Roche, Basel, Switzerland). pLX304 lentiviral vector encoding for ORF Rab5A has been described previously [33].

### 2.2. Production of Virus-Packaged shRNA Library, Transduction, and shRNA Screen

Human lentiviral shRNA library (Sigma-Mission) available at the LJI Functional Genomics Core facility was generated by the RNAi Consortium (TRC) [34,35]. shRNA clones (stored as frozen Escherichia coli glycerol stocks) were collected from master plates using Beckman Biomek FXp liquid handler and amplified in LB medium in deep-well 96-well plates (Corning, New York, NY, USA). Four individual shRNA constructs for each target gene were combined into gene-specific pools at the stage of bacterial culture (after normalization by optical density). The normalized culture was used for plasmid DNA purification (assuming equivalent DNA yields for each construct) using GenElute HP 96 Well Plasmid Miniprep kit (Sigma-Aldrich) according to the manufacturer’s protocol. Lentiviral stocks harboring pooled shRNAs were produced in HEK293T cells cultured in 24-well plates at 50–70% confluency on the day of transfection. Cells were transfected with plasmid DNA mixture containing 75 ng shRNA plasmid and 37.5 ng Sigma Mission lentiviral packaging mix per well using JetPRIME transfection reagent (Polyplus Transfection, Illkirch-Graffenstaden, France). In some experiments, 75 ng of 3:1 (psPAX2:pMD2.G) lentiviral packaging mix was used instead of the Sigma Mission packaging plasmids. The psPAX2 and pMD2 plasmids were obtained from Addgene (Cambridge, MA, USA). Viral supernatants were collected at days 2 and 3 post-transfection, filtered through a 0.45 µm low protein-binding filter, aliquoted, and stored at −80 °C. Titers of pooled shRNA viruses were measured using p24 antigen ELISA kit (ZeptoMetrix Corporation, Buffalo, NY, USA) according to the manufacturer’s protocol.

Prior to transduction, viral stocks were diluted to 2 × 10^6^ Titer Units (TU) per mL, and 100 µL aliquots of each viral preparation were used to infect 4 × 10^4^ CEM.CCR5 cells/well in a 96-well plate in the presence of 8 µg/mL polybrene (Sigma-Aldrich). Virus and cells (in 200 µL transduction volume) were centrifuged at 800× *g* for 1 h at 37 °C, and incubated at 37 °C, 5% CO_2_ for 14–17 h before changing the medium. The cells were transferred to U-bottom 96-well tissue-culture plates (Corning), centrifuged at low-speed, and the virus-containing medium was replaced with growth medium containing 1.5 µg/mL puromycin. The cells were then transferred to 24-well tissue-culture plates (Corning) and grown in the presence of puromycin for 6 days.

Measurements of HIV-1 fusion with target CEM.CCR5 harboring the shRNA cells were carried out using the β-lactamase (BlaM) assay, as described previously [11,14]. Quadruplicate aliquots of ~1.5 × 10^5^ cells/well were added to poly-l-lysine-coated 96-well Costar black clear bottom plates (Corning) and allowed to attach for 30 min at 37 °C, 5% CO_2_. Unbound cells were removed, and the plates were blocked with 100 μL/well of growth medium for 15 min at 37 °C, 5% CO_2_. HIV-1 HXB2 pseudotyped viruses bearing the BlaM-Vpr chimera (MOI = 2) were bound to cells by centrifugation at 2095× *g*, 4 °C for 30 min. To block HXB2 Env-mediated fusion, the well-characterized small molecule CXCR4 antagonist AMD3100 [36] was added to control wells. Unbound virus was washed off and fusion was initiated by shifting/incubating the samples to 37 °C, 5% CO_2_ for 90 min, after which time, cells were loaded with the fluorescent CCF4-AM substrate (Invitrogen). Intracellular BlaM activity (ratio of blue to green fluorescence) was measured using the PerkinElmer Envision microplate reader after cell incubation overnight at 12 °C. Z’ and signal-to-background (S/B) ratios were calculated as follows: Z’ = 1 − (3 × SD_control_ + 3 × SD_background_)/(F_control_ − F_background_), and S/B = F_control_/F_background_, where F is fluorescence, control corresponds to infected cells harboring Scr shRNA, and background corresponds to uninfected Scr shRNA cells.

### 2.3. Pseudovirus Production and Characterization

Pseudoviruses used in the validation assays were produced by transfection of HEK 293T/17 cells with the following amounts of plasmids per 100-mm dish: 3 μg of Env, 4 μg of pR8ΔEnv, 2 μg of BlaM-Vpr, and 0.5 μg pcRev. For the lentiviral stocks harboring either pooled shRNAs or ORF-Rab5A, cells were transfected with 4 µg pooled shRNA plasmids or ORF-Rab5A, 3 µg psPAX2, and 1.5 µg pMDG-VSVG plasmid. Transfections were carried out using JetPrime (Polyplus) transfection reagent. Forty-eight hours post-transfection, supernatants were collected, filtered through 0.45 µm filters, aliquoted, and stored at −80 °C. For fusion-from-without experiments, the viruses were concentrated by using the LentiX reagent (Clontech Laboratories, Mountain View, CA, USA) according to the manufacturer’s recommendations. The infectious units (IU) were determined either by a β-Gal assay [37], or by puromycin for shRNAs or blasticidine for ORF-Rab5A antibiotic selection in TZM-bl cells.

### 2.4. Virus-Cell Fusion (BlaM) and Infectivity Assays

For the validation BlaM experiments, triplicate aliquots of ~1.5 × 10^5^ cells/well in U-bottom 96-well plate were centrifuged at 800× *g* for 5 min at 4 °C to pellet the cells. The medium was removed and virus (MOI = 2) was added. Virus and cells (in 50 µL final volume) were centrifuged at 1550× *g* for 30 min at 4 °C. Unbound virus was washed off, 50 μL/well of growth medium was added, and fusion was initiated by incubation at 37 °C, 5% CO_2_ for 90 min. Samples were centrifuged at 800× *g* for 5 min at 4 °C to pellet the cells, medium was removed, the BlaM substrate was added and cells were transferred to poly-l-lysine coated black-clear bottom 96-well plates. Intracellular β-lactamase (BlaM) activity (ratio of blue to green fluorescence) was measured using the Synergy HT fluorescence microplate reader (Bio-Tek, Winooski, VT, USA) following an overnight incubation at 12 °C.

For the infectivity assays, triplicate aliquots of ~2.5 × 10^4^ cells/well in U-bottom 96-well plate and virus (MOI = 0.5) were centrifuged at 1550× *g* for 30 min at 4 °C. Unbound virus was washed off, 75 μL/well of growth medium was added, samples were transferred into black-clear bottom 96-well plates, and incubated at 37 °C, 5% CO_2_. Forty-eight hours post-infection, equal volume of Bright-Glo^TM^ firefly luciferase substrate (Promega, Madison, WI) was added, samples were incubated for 5 min at room temperature, and the resulting luciferase signal was measured using a TopCount NXT plate reader (PerkinElmer Life Sciences, Waltham, MA, USA).

### 2.5. Fusion-From-Without Assay

To measure fusion-from-without (FFWO) between CEM.CCR5/shRNA cells, cells were suspended in OPTI-MEM. One-half of cells were labeled with 2 μM CellTracker^TM^ Orange (CMRA), while the second half was loaded with 1 μM CellTracker^TM^ Green (CMFDA). Differently labeled cells were washed to remove residual dye, mixed at a 1:1 ratio, and transferred to a U-bottom 96-well plate (1.5 × 10^5^ cells/well). Viruses (MOI = 10) were bound to cells by centrifugation at 1550× *g* at 4 °C for 30 min. Unbound virus was removed, and the samples were incubated in a growth media for 2 h at 37 °C, 5%CO_2_. The cells were then placed on ice, washed with cold PBS, suspended in live-cell imaging buffer/2% FBS, and adhered to poly-l-lysine-coated 8-chamber cove slips (Lab-Tek, Nunc, Waltham, MA, USA) for 10 min at 4 °C prior to imaging on an Olympus IX-71 microscope equipped with an EM-CCD camera (Hamamatsu, C9100-12, Shizuoka, Japan). The fraction of effector and target cells that fused was determined by counting the number of cells positive for both dyes using ImageJ software (National Institutes of Health, Bethesda, Rockville, MD, USA).

### 2.6. Virus Uptake Assay

To assess virus internalization by CEM cells, virus-cell complexes formed in the cold, as described above, were incubated at 37 °C, 5% CO_2_ for 60 min. After the incubation step, uninternalized viruses were removed by incubating with 0.25% trypsin (Sigma) for 5 min at room temperature. Following an extensive washing step with growth medium, cells were lysed and subjected to p24 ELISA analysis, as described previously [38]. The efficiency of virus uptake was determined by normalizing to the p24 signal following the trypsin-stripping of viruses of control cells maintained at 4 °C.

### 2.7. Gain-of-Function Experiments

CEM.CCR5 cells were infected with lentiviruses carrying shRab5A specific for 3’-UTR (MOI = 3) by centrifugation, as described above, and selected with 1.5 µg/mL puromycin for 36 h. Selected cells were either left untreated or were transduced with lentiviruses harboring ORF-Rab5A (MOI ~ 5) and selected with 16 µg/mL blasticidin for additional 36 h.

### 2.8. Western Blotting and Flow Cytometry

For Western blotting, the extracts of CEM.CCR5/shRNA were prepared using RIPA buffer without SDS (50 mm Tris-HCl (pH 7.4), 1% Nonidet P-40, 0.1% sodium deoxycholate, and 150 mM NaCl) supplemented with protease inhibitors (Roche Applied Science), followed by centrifugation at 1500× *g* for 5 min to sediment nuclei. Extracts were adjusted to equivalent protein concentrations, using the BCA protein assay kit (Thermo Fisher Scientific, Waltham, MA, USA). Protein content-adjusted samples were loaded onto 4–15% SDS-PAGE (Bio-Rad, Hercules, CA, USA), followed by transfer onto a nitrocellulose membrane that was pretreated with 10% Blotting-grade Blocker (Bio-Rad) for 30 min at room temperature. The membrane was next incubated with the primary antibodies prepared in 5% Blotting-grade Blocker overnight at 4 °C. Horseradish peroxidase-conjugated (HRP) antibodies and a chemiluminescence reagent from GE Healthcare (Chicago, IL, USA) were used for protein detection. Precision Plus protein standards (Kaleidoscope^TM^, Bio-Rad) were used as molecular weight markers.

To assess the HIV-1 receptor levels, cells were harvested by low speed centrifugation, washed and incubated for 2 h in the cold with primary mouse anti-hCD4 APC-conjugated (SK3 clone) antibody and the anti-CXCR4 APC-conjugated 12G5 antibody (both from eBioscience, Santa Clara, CA, USA) diluted 1:1000 in PBS/2% FBS. A mouse anti-CCR5 antibody (1:200, BD Pharmingen, San Diego, CA, USA) and a sheep anti-mouse FITC-labeled antibody (1:1000, Sigma-Aldrich) were used to assess CCR5 expression. Samples were measured on a BD LSR II FACS system (BD Biosciences, San Jose, CA, USA) and analyzed using FlowJo software (LLC, Ashland, OR, USA).

## 3. Results

### 3.1. Screen for Endosomal Factors Involved in HIV-1 Fusion with CD4+ T Cells

**Figure 1:**
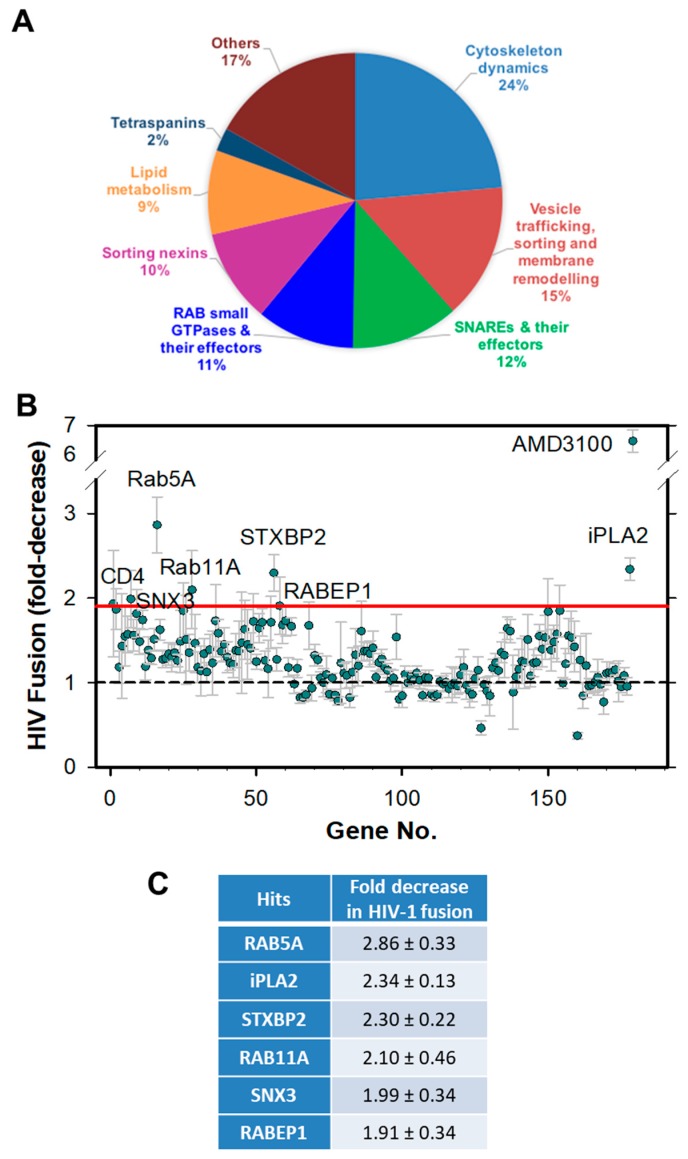
Targeted shRNA screen for factors facilitating HIV-1 fusion with CEM.CCR5 cells. (**A**) A pie-chart representation of the main gene families targeted by the shRNA screen. (**B**) Results of HXB2 pseudovirus-cell fusion (BlaM) assay using cells depleted of selected proteins. The results are plotted as fold-decrease in the fusion signal compared to that with cells transduced with a scrambled shRNA. The red horizontal line indicates the cut-off used to select hits. The data points are means and SD from quadruplicate wells. (**C**) A table showing the hits and the fold-decrease in HXB2 pseudovirus fusion.

To identify endosomal factors that modulate HIV-1 fusion with CD4+ T lymphocytes, we performed a targeted shRNA screen using the human CEM.NKR-CCR5.Luc cell line (hereafter referred to as CEM.CCR5) that stably expresses CCR5 and CXCR4 and contains a firefly luciferase expression cassette under a Tat-inducible promoter [26]. Our screening strategy differed from the previous screens for HIV-1 host factors in two important aspects. First, we employed a direct virus-cell fusion assay as readout, as opposed to the single-cycle and multi-cycle infection assays (e.g., [24]). This is significant, because the effects on virus entry/fusion are more difficult to assess using infectivity assays that rely on completion of multiple post-fusion steps which in turn can be affected by protein knock-down. Second, since a large fraction of hits obtained by the previous genome-wide RNAi screens were factors involved in transcriptional regulation, nuclear transport, biosynthesis and signaling pathways that are unlikely to affect HIV-1 fusion, we focused on proteins involved in endocytosis, membrane trafficking, and lipid metabolism (Figure 1A).

We used four shRNAs per gene selected from a human lentiviral shRNA library (Sigma-Mission) cloned into the PLK0.1 expression vector [34,35]. In order to achieve highly efficient transduction of CEM.CCR5 cells with lentiviral vectors encoding the shRNAs, we used virus-like particles pseudotyped with the Vesicular Stomatitis Virus G glycoprotein (VSV-G). Following the shRNA transduction, cells were subjected to puromycin selection for four to six days. HIV-1 fusion with CEM.CCR5/shRNA cells was examined using the β-lactamase (BlaM) assay that measures the extent of viral fusion based on the cytosolic β-lactamase activity [14,39]. Briefly, quadruplicate samples of CEM.CCR5/shRNA cells were attached to poly-l-lysine treated 96-well plates. HIV-1 pseudoviruses bearing the BlaM-Vpr reporter and HXB2 (X4-tropic) Env were pre-bound to target cells in the cold, and virus-cell fusion was initiated by shifting samples to 37 °C and incubating for 90 min. At the end of incubation, cells were loaded with a fluorescent BlaM substrate and incubated overnight at 12 °C to allow substrate cleavage by β-lactamase but prevent further fusion events. This protocol yielded a signal/background (S/B) ratio of 10 and a Z’ value of 0.7 (maximum value is 1), indicating robust assay performance for a screen.

Our shRNA screen targeted 195 genes whose products are involved in various endocytic functions, such as cytoskeleton dynamics, vesicle trafficking, sorting and membrane remodeling (Figure 1A and Table S1). The resulting extents of HIV-1 fusion were normalized to the values measured in control cells transduced with scrambled (Scr) shRNA. shRNAs specific for CD4 and CXCR4 were used as positive controls. Among the 195 targeted genes, knockdown of 18 genes proved lethal to cells. A relatively low fraction of non-viable clones, as compared to the previous screen that employed similar cells [24], is likely due to the shorter puromycin selection time after shRNA transduction in our experiments.

**Figure 2:**
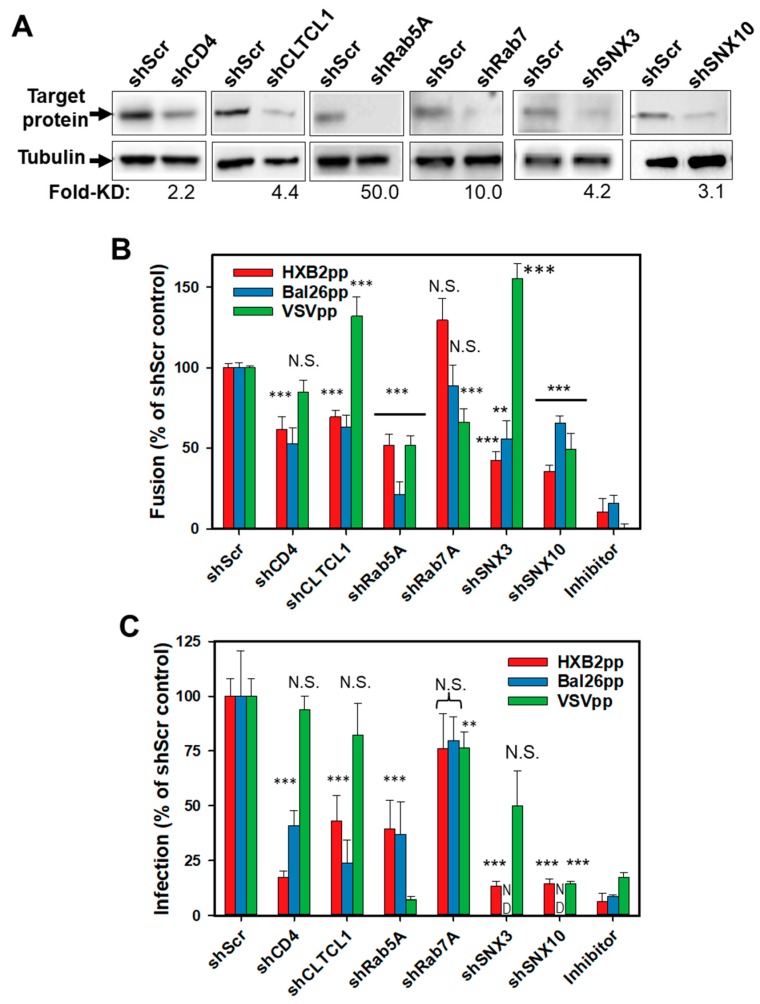
Validation of hits from the shRNA screen. (**A**) Western blot analysis of protein expression levels in control cells (shScr) and cells transduced with indicated shRNAs. Tubulin bands are loading controls. Fold-decrease in the protein expression level in shRNA-transduced cells relative to control cells determined by densitometry analysis is shown below the image panels. (**B**) Fusion activity of HXB2pp, BaL26pp and VSVpp in CEM.CCR5.shRNA cells relative to CEM.CCR5.shScr control cells. (**C**) Infectivity of HXB2pp, BaL26pp and VSVpp shRNA-transduced cells relative to control cells. As negative controls, HIV-1pp or VSVpp fusion experiments were carried out in the presence of 1 µM C52L peptide or 30 mM NH_4_Cl, respectively. For shCD4, shCLTCL1, shRab5A and shRab7A the data points in B and C are means and SD from three independent experiments performed in triplicate. For shSNX3 and shSNX10 the data points in B and C are means and SD from two independent triplicate experiments. **, *P* < 0.01; ***, *P* < 0.001; NS, not significant.

Knockdown of either CD4 or CXCR4 resulted in a ~1.9-fold decrease in HIV-1 fusion (Figure 1B and Table S1). This partial inhibition of HIV-1 fusion was likely due to a modest depletion of these proteins by specific shRNAs (see Figure 2 below). Considering such a modest effect of knockdown of the HIV-1 cellular receptors, positive hits were defined as shRNA clones exhibiting ≥1.9-fold decrease in the extent of fusion. Six hits were identified using this cut-off, yielding a 3% hit rate from the screen. These hits included two Rab GTPases, Rab5A and Rab11A, the Rab effector protein, RABEP1, sorting nexin 3 (SNX3), the SNARE effector, syntaxin binding protein 2 (STXBP2), and the Ca^2+^-independent phospholipase A2 (iPLA2) (Figure 1B,C). Knockdown of Rab5A, a key player in early endosome biogenesis, or SNX3, which is involved in membrane trafficking, resulted in a robust ~2.9-fold and 2.0-fold decrease in HIV-1 fusion, respectively (Figure 1C). Notably, STXBP2 and Rab11A have been previously identified as HIV-1 dependency factors in CEM cells by a genetic screen [24].

### 3.2. Clathrin-Mediated Endocytosis, Rab5A- and Sorting Nexin-Dependent Processes Contribute to Productive HIV-1 Entry in CEM Cells

Next, we sought to validate the selected hits from the screen by measuring the fusion activity and infectivity of HIV-1 particles pseudotyped with two Env strains or with VSV-G in shRNA-transduced cells. Three additional host factors not included in the original screen were also tested. Considering that clathrin-mediated endocytosis is a major HIV-1 uptake pathway in HeLa and CD4+ T cells [40,41], we expanded our analysis of HIV-1 host factors in CEM cells using shRNA to the heavy chain of clathrin (CLTCL1). In addition, encouraged by the finding that cells carrying shRNA to SNX3 are less permissive to HIV-1 fusion than control cells, we examined the role of a related sorting nexin, SNX10, which has been identified in the previous genetic screen for HIV-1 factors in Jurkat cells [24]. We also tested the efficiency of HIV-1 fusion with cells depleted of Rab7A, a regulator of late endosome biogenesis. We generated CEM.CCR5 knockdown cell lines using 5 discrete shRNAs/gene-targeting independent sequences within a given mRNA (Sigma). Scrambled shRNA and shRNA to CD4 were included as negative and positive controls, respectively. Western blotting confirmed successful depletion of target proteins by specific shRNAs (Figure 2A). The only exception was CD4, which exhibited significant residual expression in cells harboring shCD4.

The shRNA-transduced clones were tested for their ability to support fusion of pseudoviruses bearing HXB2 Env (HXB2pp), CCR5-tropic BaL26 Env (BaL26pp) or an unrelated VSV-G protein (VSVpp) that mediates low pH-dependent virus entry through an endocytic pathway. Cells infected in the presence of either the HIV-1 fusion inhibitory C52L peptide [28] or NH_4_Cl, which blocks VSV-G mediated fusion by raising the endosomal pH, were used as negative controls. A relatively modest, but significant decrease in the HXB2pp and BaL26pp fusion was observed in shCD4 transduced cells (Figure 2B and also Figure 1B), consistent with the inefficient knockdown of CD4 in these cells (~50% of the control cell level, Figure 2A). HXB2pp and BaL26pp fused less efficiently with cells harboring shCLTCL1, supporting the role of clathrin-mediated endocytosis in productive HIV-1 entry into CEM cells. Importantly, Rab5A knockdown inhibited HXB2pp and BaL26pp fusion, whereas silencing Rab7A was without effect (Figure 2B). Depletion of SNX3 or SNX10 also decreased the fusion efficiency of HXB2pp and BaL26pp. These results imply that the functions of early endosome-resident Rab5A protein and the sorting nexins 3 and 10 are involved in HIV-1 entry/fusion, irrespective of coreceptor tropism.

The diminished efficiency of HIV-1 fusion with cells harboring shRNAs to clathrin heavy chain, Rab5A, SNX3, or SNX10 was not due to downregulation of CD4 or coreceptor expression in these cells. Flow cytometric analysis showed no significant differences in the levels of CD4, CXCR4, and CCR5 expression in control cells and cells transduced with the above shRNAs (Figure S1A,C). The only exception was the reduced level of CCR5 expression on the surface of CEM.CCR5 cells transduced with shRNA to Rab5A (Figure S1A). However, these cells ectopically express high levels of CCR5 and a modest reduction in surface expression is unlikely to considerably affect HIV-1 fusion. Besides, the diminished CCR5 expression does not account for the highly significant inhibition of HXB2pp fusion with Rab5A-depleted cells (Figure 1B and Figure 2B). Neither of the puromycin-selected cell clones harboring shRNAs to clathrin heavy chain, Rab5A, SNX3 or SNX10 exhibited reduced viability (Figure S1B,D).

As expected, VSVpp fusion was not affected by CD4 depletion, but was inhibited in Rab5A- and SNX10-depleted cells and enhanced upon SNX3 knockdown (Figure 2B). The opposite effect of SNX3 depletion on HIV-1 and VSVpp fusion (Figure 2B) is notable, as this result argues against a non-specific effect of SNX3 knockdown on the overall endocytic and protein trafficking activities. Surprisingly, Rab7A knockdown in CEM.CCR5 cells also reduced the VSVpp fusion efficiency. This result is in apparent disagreement with the previously reported VSV fusion with early endosomes in adherent cell lines (e.g., [42,43,44]). Another surprising finding was the enhancement of VSVpp fusion in cells depleted of CLTCL1 (Figure 2B). This finding suggests that VSV-G can promote infection of CEM cells via a clathrin-independent pathway. Collectively, these results indicate that the VSV entry pathway is cell type-dependent and is distinct from the endocytic entry pathway of HIV-1.

We next assessed the effects of protein knockdown on HIV-1 infectivity in CEM.CCR5 cells using a single-cycle infectivity assay that measures luciferase expression. In general agreement with the fusion results (Figure 2B), HXB2pp and BaL26pp infection of CEM.CCR5 cells harboring shRNAs to selected cellular factors was diminished compared to shScr-transduced cells (Figure 2C). On average, the effect of candidate protein depletion on HIV-1 infectivity tended to be stronger than on virus-cell fusion (compare Figure 2B,C). VSVpp infectivity was markedly inhibited by knockdown of Rab5A, SNX10 and, to a lesser extent, by Rab7A (Figure 2C), consistent with effects on fusion. Although depletion of CLTCL1 and SNX3 promoted the VSVpp fusion, no significant effects on infection were observed.

**Figure 3:**
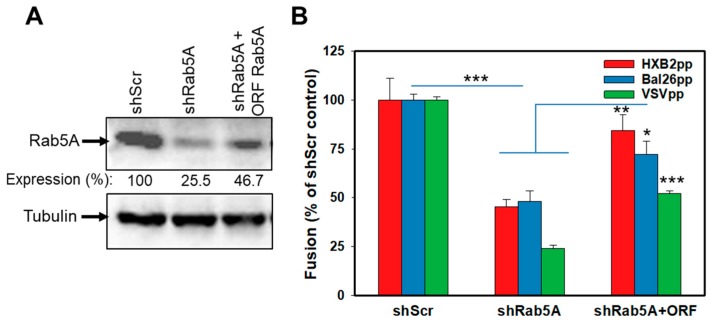
Partial rescue of HIV-1 and VSV fusion with shRNA-transduced cells upon ectopic expression of Rab5A. CEM.CCR5 cells transduced with Rab5A shRNA were transduced with a vector expressing shRNA-resistant Rab5A construct. (**A**) Western blot analysis of Rab5A expression. Rab5A expression levels relative to control cells determined by densitometry analysis are shown below the top image panel. (**B**) HXB2pp, BaL26pp and VSVpp fusion activity, as measured by the BlaM assay, is enhanced upon expression of Rab5A in Rab5A-depleted cells. The data points are means and SEM from two independent triplicate experiments. *, *P* < 0.05; **, *P* < 0.01; ***, *P* < 0.001.

Next, we performed the gain-of-function experiments to control against possible off-target effects of shRNA knockdown. We selected the *rab5A* gene and used an Rab5A-ORF construct that lacks the 3′-untranslated region targeted by shRNA. The introduction of Rab5A-ORF partially restored the Rab5A expression in Rab5A-depleted cells, as assessed by Western blotting (Figure 3A). Importantly, ectopic expression of Rab5A in shRab5A cells significantly rescued the HXB2pp, BaL26pp and VSVpp fusion, albeit not quite to the level observed in the control shScr cells (Figure 3B). Such incomplete rescue of the BlaM signal is most likely due to a partial recovery (up to approximately half of the control Rab5A expression level, Figure 3A) of the Rab5A expression in shRab5A-transduced cells.

The inhibition of HIV-1 fusion and infection in cells depleted of Rab5A, SNX3 or SNX10 suggests that this virus enters CEM.CCR5 cells via an endocytic pathway. However, alternative explanations to these findings are possible. Knockdown of the above factors may: (1) accelerate virus uptake, thereby disfavoring fusion at the cell surface and targeting HIV-1 for lysosomal degradation and/or (2) interfere with HIV-1 fusion with the plasma membrane through an unknown mechanism. To address these possibilities, we performed additional experiments described below.

### 3.3. Factors Involved in HIV-1 Entry/Fusion do not Considerably Affect Virus Uptake

**Figure 4:**
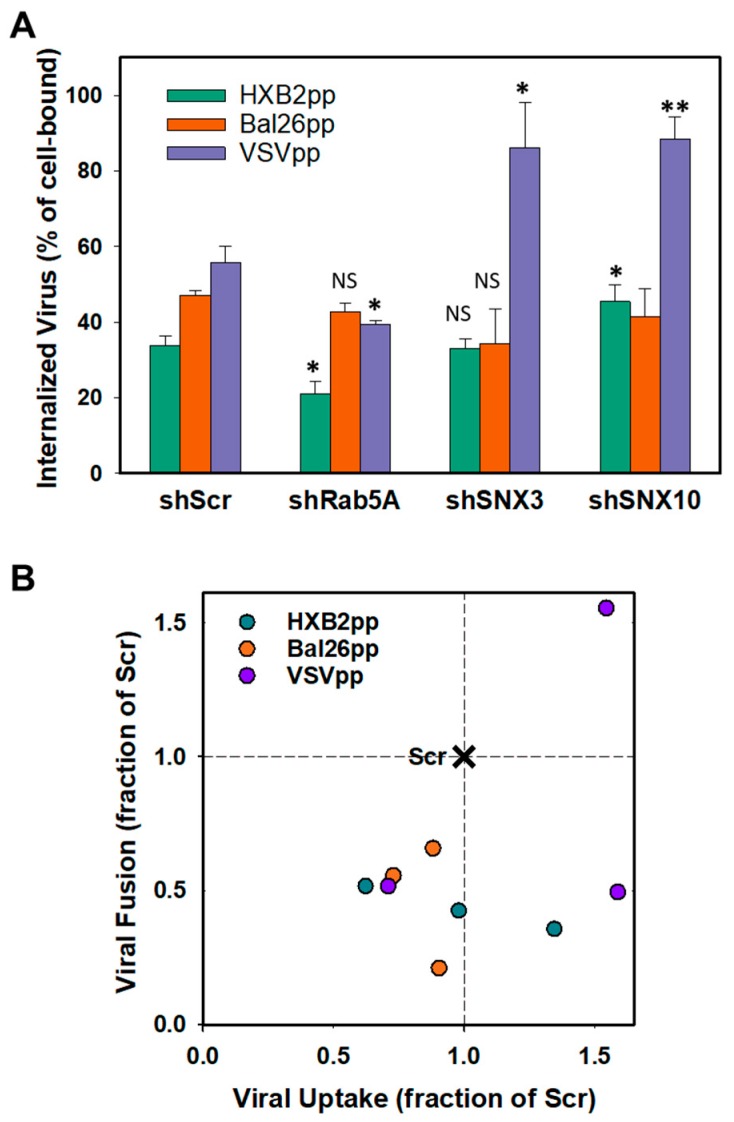
Effect of protein knockdown on virus uptake by cells. (**A**) Rab5A, SNX3 and SNX10 were knocked down in CEM.CCR5 cells and the ability of shRNA-transduced cells to internalize HXB2pp, Bal26pp or VSVpp was measured after stripping of cell surface-associated virus with trypsin 1 h post-inoculation (see Methods). The fraction of internalized virus for each condition is plotted. The data points are means and SEM from two independent triplicate experiments. *, *P* < 0.05; **, *P* < 0.01; NS, not significant. (**B**) Correlation between virus uptake by and fusion with CEM.CCR5 cells depleted of Rab5A, SNX3 or SNX10 (data from Figure 2B and Figure 4A) plotted as fractions of the shScr control.

We next asked if depletion of Rab5A, SNX3 or SNX10 in CEM.CCR5 cells inhibits HIV-1 fusion and infection by modulating the virus uptake. Virus endocytosis was measured using a protease accessibility assay described previously [13,45]. Briefly, after a 1 h-incubation with viruses at 37 °C, cells were washed, surface-accessible virions were removed by trypsin treatment and the remaining p24 amounts measured by ELISA. Whereas BaL26pp uptake was not affected by knockdown of either of the three proteins, HXB2pp internalization was modestly decreased or increased in cells depleted of Rab5A or SNX10, respectively (Figure 4A). We found that VSVpp uptake was also slightly reduced in Rab5A-depleted cells, but was potently enhanced in sorting nexin-depleted cells (Figure 4A). Importantly, the effects of Rab5A and sorting nexin knockdown on HIV-1 or VSV pseudovirus uptake did not correlate with the effects on virus-cell fusion (Figure 4B). The only exception was the enhanced VSVpp uptake and fusion in CEM.CCR5 cells depleted of SNX3. Note, however, that the increased VSVpp fusion with SNX3 depleted cells was not associated with enhanced infectivity (Figure 2).

The lack of correlation between the VSVpp uptake and fusion shows that endocytosis is necessary but not sufficient for efficient virus fusion with endosomes. Our result thus highlights the importance of post-uptake cellular processes for efficient virus entry and the importance of discerning between bulk virus uptake and productive entry. We thus concluded that bulk HIV-1 or VSV-G pseudovirus uptake, which enters cells through endocytosis, does not generally correlate with viral fusion (Figure 4B) or infection (compare Figure 2C and Figure 4A). Our results also show that the inhibition of HIV-1 fusion with CEM.CCR5 cells depleted of the above host factors is not caused by accelerated uptake that could target the virus to degrading compartments.

### 3.4. Endocytic Factors Involved in Productive HIV-1 Entry Do Not Affect the Virus’s Ability to Undergo Fusion with the Plasma Membrane

**Figure 5:**
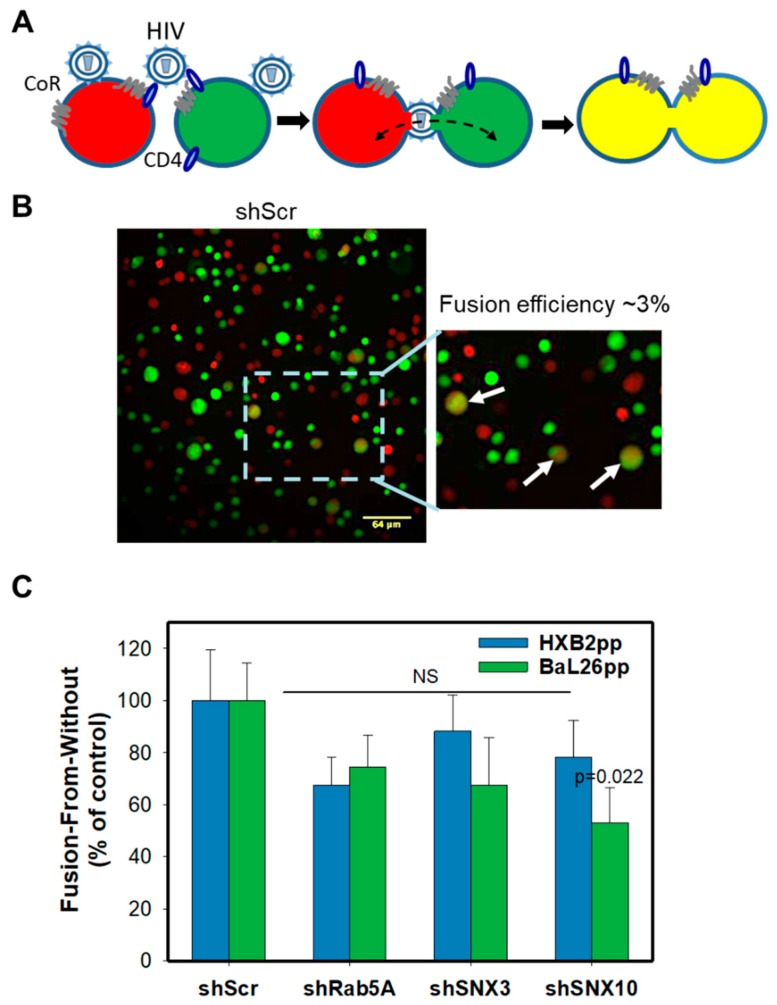
Effect of protein knockdown on HIV-1 mediated cell-cell fusion. (**A**) A depiction of the “fusion-from-without” (FFWO) assay that is based on detection of cell-cell fusion mediated by HIV-1 virions attached to adjacent cells. CoR, coreceptor. Cells are loaded with green (CMFDA) or orange/red (CMRA) cytoplasmic dyes and virus-mediated fusion between two adjacent cells is detected by appearance of double-labeled fusion products. (**B**) Representative images of fusion (FFWO) between scrambled shRNA-transduced CEM.CCR5 cells mediated by HXB2pp. Insert shows an enlarged view of the boxed area; the fused cells are marked by arrows. (**C**) Quantification of HXB2pp- and Bal26pp-mediated cell-cell fusion. The data points are mean and S.E. of 20 images from two independent experiments.

The requirement for Rab5A, SNX3 and SNX10 for efficient HIV-1 fusion and infection in CEM.CCR5 cells suggests that endocytosis plays a role in productive entry into these cells. However, it remained possible that although the levels of CD4 or coreceptor expression in CEM.CCR5 cells were not significantly reduced (Figure S1), depletion of Rab5A or sorting nexins could compromise the virus’s ability to fuse with the plasma membrane. To assess this possibility, we employed a fusion-from-without (FFWO) assay [46], which monitors cell-cell fusion mediated by viral particles fusing to the plasma membranes of two adjacent cells [13] (Figure 5A). FFWO offers a convenient means to isolate HIV-1 fusion events occurring at the cell surface from those that may be occurring in endosomes. We have previously used this assay to demonstrate that unlike HIV-1-cell fusion via a regular route, FFWO is remarkably inefficient and is highly dependent on actin dynamics [13]. Thus, the measurements of FFWO in cells transduced with the selected shRNA enable the assessment of the efficiency of rare HIV-1 fusion events occurring at the cell surface.

HIV-1-mediated cell-cell fusion was monitored by fluorescence microscopy. Briefly, two aliquots of CEM.CCR5 cells, one labeled with a green and another with an orange cytoplasmic marker, were mixed at 1:1 ratio in a 96-well U-bottom plate and centrifuged with pseudoviruses at a high MOI of 10 in the cold to facilitate cell-to-cell contacts and facilitate the virus binding to adjacent cells (Figure 5A). Samples were next incubated at 37 °C and the resulting cell-cell fusion products were deposited onto poly-lysine-coated slides for imaging. Cell-cell fusion was detected by the appearance of double-positive (green/red) cells (Figure 5A,B). The efficiency of FFWO determined by normalizing the number of double-positive CEM cells to the total number of cells per field was low (~3%), in agreement with our previous results [13]. Analysis of the FFWO results showed no significant effect of Rab5A, or SNX3 knockdown on the efficiency of HXB2pp- or BaL26pp-mediated cell-cell fusion, as compared to the shScr control (Figure 5C). The only exception were cells depleted of SNX10 that exhibited a statistically significant decrease in FFWO induced by BaL26pp, but not by HXB2pp. This finding reinforces the notion that the above endocytic factors modulate fusion of endocytosed HIV-1, while generally not affecting the ability of virions trapped between two adjacent CEM.CCR5 cells to fuse with the plasma membranes.

## 4. Discussion

The shRNA screen for host factors involved in HIV-1 fusion with CEM.CCR5 cells performed in this study revealed several proteins involved in endocytosis and membrane trafficking as essential factors for productive virus entry. Control experiments showed that depletion of these factors does not: (1) downregulate the CD4 or coreceptor expression, (2) strongly affect HIV-1 endocytosis or (3) modulate the virus’s ability to fuse with the plasma membrane of two adjacent cells. These results rule out the possibility that the selected proteins affect HIV-1 fusion indirectly, by altering the receptor/coreceptor expression or by quickly sequestering the virus into endosomes that are not conducive to fusion. Importantly, the unaltered ability of this virus to mediate cell-cell fusion argues against the possibility that depletion of Rab5A, SNX3 or SNX10 disfavors fusion at the cell surface by an unknown mechanism.

Our results thus strongly support a role of endocytic entry/fusion in HIV-1 infection of CEM cells. The diminished HIV-1 fusion and infection in cells in which the clathrin function was inhibited supports the role of clathrin-mediated endocytosis in HIV-1 entry/fusion. Furthermore, the sensitivity of HIV-1 fusion and infection to Rab5A knockdown but not to Rab7A knockdown suggests that HIV-1 may tend to fuse in early endosomes of CEM cells. The differential effects of clathrin and SNX3 knockdown on fusion driven by the HIV-1 Env and VSV-G (Figure 2B,C) indicates that these viruses enter CEM cells through distinct endocytic pathways. Note that, based on a bulk HIV-1 uptake assay, Herold and co-authors [10] have concluded that endocytosis does not contribute to HIV-1 entry into CD4 T cells. However, the lack of correlation between bulk virus uptake and fusion in CEM-derived cells, even for VSVpp (Figure 4), implies that this indirect approach may be misleading since the majority of internalized virions do not appear to establish infection. Regardless of the reasons for these discrepant findings, our protein knockdown approach suggests that endocytic transport of HIV-1 is required for optimal fusion with CEM cells. While our results do not rule out HIV-1 fusion with the plasma membrane of these cells, they are consistent with the model that virus uptake and transport to conducive compartments is essential for productive entry.

SNX3 and SNX10 are PI3P-binding proteins involved in intracellular protein trafficking (reviewed in [47,48]). SNX3 is required for multivesicular body formation and for protein transport between early and recycling compartments, as well as for transport between endosomes and the trans-Golgi network [47,49,50]. The localization of SNX3 to PI3P-enriched early/recycling endosomes is consistent with the notion that HIV-1 may fuse with early endosomes that is also supported by the Rab5A knockdown results.

It is currently unknown how Rab5A, SNX3 or SNX10 facilitate HIV-1 fusion. Future experiments will provide important mechanistic insights into the sites of HIV-1 fusion with these cells and reveal cellular processes exploited by this virus. Although live cell imaging of HIV-1 fusion with CD4+ T cells is challenging, improved virus labeling and live cell imaging techniques should enable the identification of intracellular compartments that are conducive to HIV-1 fusion.

## Supplementary Materials

The following are available online at http://www.mdpi.com/1999-4915/11/2/100/s1. Supplementary Figure S1. Effect of protein knockdown on cell surface expression of HIV-1 receptors and on cell viability. Supplementary Table S1. Results of shRNA screen for host factors involved in HIV-1 fusion.
