# Supplementary material for: HIV-1 Fusion with CD4+ T cells Is Promoted by Proteins Involved in Endocytosis and Intracellular Membrane Trafficking
Source: Viruses. 2019 Jan 25;11(2):100. doi: 10.3390/v11020100 (PMC6409670; doi:10.3390/v11020100)
Supplement: Supplementary file 1 [file viruses-11-00100-s001.zip › SupplFig1.pdf]

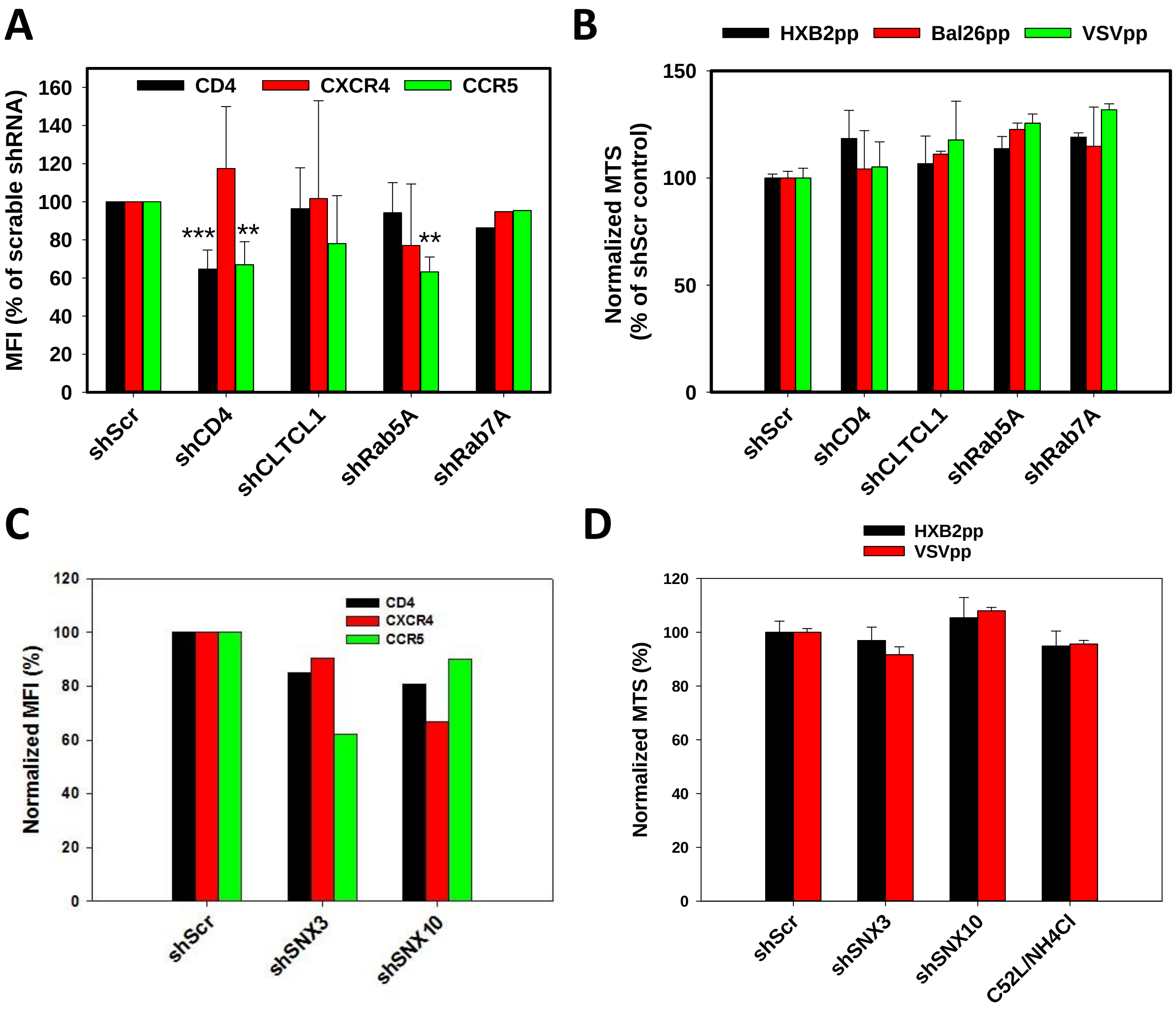

Figure S1. Effect of protein knockdown on cell surface expression of HIV-1 receptors and on cell viability. (A and C) The mean fluorescence intensity (MFI) for cell surface levels of CD4, CXCR4 and CCR5 in CEM.CCR5.shRNA clones was quantified by flow cytometry. In (A) the data points are mean and SD of four independent experiments. In (C) the data points represent one experiment. (B and D) The cell viability of the CEM.CCR5.shRNA clones infected with HXB2pp, BaL26pp or VSVpp (MOI=2) at 37°C for 90 min was measured using CellTiter 96 Aqueous Reagent (MTS) (Promega), and further incubated at 37°C for 90 min. The absorbance at 490 nm was recorded using Synergy HT fluorescence plate reader (Bio-Tek, Germany). The data points are mean and SD of two independent triplicate experiments.
